# High-dose mitoxantrone with peripheral blood progenitor cell rescue: toxicity, pharmacokinetics and implications for dosage and schedule.

**DOI:** 10.1038/bjc.1997.465

**Published:** 1997

**Authors:** A. Ballestrero, F. Ferrando, A. Garuti, P. Basta, R. Gonella, M. Esposito, M. O. Vannozzi, G. Sorice, D. Friedman, M. Puglisi, F. Brema, G. S. Mela, M. Sessarego, F. Patrone

**Affiliations:** Dipartimento di Medicina Interna, UniversitÃ di Genova, Genoa, Italy.

## Abstract

The optimal use of mitoxantrone (NOV) in the high-dose range requires elucidation of its maximum tolerated dose with peripheral blood progenitor cell (PBPC) support and the time interval needed between drug administration and PBPC reinfusion in order to avoid graft toxicity. The aims of this study were: (1) to verify the feasibility and haematological toxicity of escalating NOV up to 90 mg m(-2) with PBPC support; and (2) to verify the safeness of a short (96 h) interval between NOV administration and PBPC reinfusion. Three cohorts of ten patients with breast cancer (BC) or non-Hodgkin's lymphoma (NHL) received escalating doses of NOV, 60, 75 and 90 mg m(-2) plus melphalan (L-PAM), 140-180 mg m(-2), with PBPC rescue 96 h after NOV. Haematological toxicity was evaluated daily (WHO criteria). NOV plasma pharmacokinetics was also evaluated, as well as NOV cytotoxicity against PBPCs. Haematological recovery was rapid and complete at each NOV dose level without statistically significant differences, and there were no major toxicities. NOV plasma concentrations at the time of PBPC reinfusion were below the toxicity threshold against haemopoietic progenitors. It is concluded that, when adequately supported with PBPCs, NOV can be escalated up to 90 mg m(-2) with acceptable haematological toxicity. PBPCs can be safely reinfused as early as 96 h after NOV administration.


British Journal of Cancer (1997) 76(6), 797–804
© 1997 Cancer Research Campaign

### High-dose mitoxantrone with peripheral blood progenitor cell rescue: toxicity, pharmacokinetics and implications for dosage and schedule

**A Ballestrero[1], F Ferrando[1], A Garuti[1], P Basta[1], R Gonella[1], M Esposito[2], MO Vannozzi[2], G Sorice[3], D Friedman[3], M Puglisi[4], F Brema[5], GS Mela[1], M Sessarego[1] and F Patrone[1]**

[1]Dipartimento di Medicina Interna, Università di Genova, Genoa, Italy; [2]Istituto Nazionale per la Ricerca sul Cancro, Genoa, Italy; [3]Clinica Chirurgica B, Università di Genova, Genoa, Italy; [4]Clinica Chirurgica R, Università di Genova, Genoa, Italy; [5]Servizio di Oncologia Medica, Ospedale San Paolo, Savona, Italy

**Summary** The optimal use of mitoxantrone (NOV) in the high-dose range requires elucidation of its maximum tolerated dose with peripheral blood progenitor cell (PBPC) support and the time interval needed between drug administration and PBPC reinfusion in order to avoid graft toxicity. The aims of this study were: (1) to verify the feasibility and haematological toxicity of escalating NOV up to 90 mg $m^{-2}$ with PBPC support; and (2) to verify the safeness of a short (96 h) interval between NOV administration and PBPC reinfusion. Three cohorts of ten patients with breast cancer (BC) or non-Hodgkin's lymphoma (NHL) received escalating doses of NOV, 60, 75 and 90 mg $m^{-2}$ plus melphalan (L-PAM), 140–180 mg $m^{-2}$, with PBPC rescue 96 h after NOV. Haematological toxicity was evaluated daily (WHO criteria). NOV plasma pharmacokinetics was also evaluated, as well as NOV cytotoxicity against PBPCs. Haematological recovery was rapid and complete at each NOV dose level without statistically significant differences, and there were no major toxicities. NOV plasma concentrations at the time of PBPC reinfusion were below the toxicity threshold against haemopoietic progenitors. It is concluded that, when adequately supported with PBPCs, NOV can be escalated up to 90 mg $m^{-2}$ with acceptable haematological toxicity. PBPCs can be safely reinfused as early as 96 h after NOV administration.

**Keywords:** high-dose chemotherapy; mitoxantrone; peripheral blood progenitor cells

High-dose chemotherapy is commonly based on alkylating agents, mainly because of the myeloid dose-limiting toxicity, the steep dose–response curve in vitro and the favourable dose ratio (with a four- to tenfold escalation with respect to conventional doses) of these drugs (Frei and Canellos, 1980; Frei, 1995). However, the growing extension of high-dose chemotherapy to solid tumours and the development of multistep high-dose programmes have led to a broader use of drugs belonging to different classes and acting by different mechanisms (Gianni and Bonadonna, 1989).

Among the non-alkylating agents, mitoxantrone (NOV), an anthraquinone compound that is active against a variety of haematological and solid tumours, has received considerable attention. In fact, NOV has haematological dose-limiting toxicity with reportedly limited cardiotoxicity compared with other anthracyclines, can be escalated at least five times above the conventional dose and clearly exhibits a steep dose–response curve in vitro (Von Hoff et al, 1986). Thus, it has been included in several high-dose regimens delivered with haematopoietic rescue (Mulder et al, 1989; Ellis et al, 1990; Wallerstein et al, 1990; Bowers et al, 1993; Attal et al, 1994; Stiff et al, 1994; Patrone et al, 1995). However, it has been claimed that very high dosages imply haematological toxicity that cannot be adequately rescued even by haematopoietic progenitor cell reinfusion (Attal et al, 1994). A special matter of concern arises from its prolonged plasma half-life and possible toxicity to reinfused progenitors, especially at higher dosages. This is why a prolonged interval between NOV administration and haematopoietic rescue is often recommended, which may involve some disadvantages, mainly because delaying reinfusion delays haematological recovery.

In this study we tested the feasibility of administering NOV safely with peripheral blood progenitor cell (PBPC) support in the dose range 60–90 mg $m^{-2}$, and we also addressed the issue of haematopoietic rescue timing. For these purposes, clinical and pharmacokinetic evaluations were carried out in patients undergoing high-dose treatments including NOV at dosages of 60, 75 and 90 mg $m^{-2}$. Furthermore, the cytotoxicity threshold of the drug against haematopoietic progenitor cells was determined by in vitro experiments.

*Received 10 December 1996*
*Revised 5 March 1997*
*Accepted 11 March 1997*

*Correspondence to:* F Patrone, DIMI Università, Viale Benedetto XV n. 6, 16132 Genova, Italy

### MATERIALS AND METHODS

### Study design

Thirty consecutive patients eligible for high-dose chemotherapy programmes including a conditioning regimen with NOV and melphalan (L-PAM) were enrolled in the present study. In order to assess haematological and non-haematological toxicity of escalating doses of NOV, administered at supposedly myeloablative doses with PBPC rescue, three cohorts of ten patients each were treated at the dose levels of 60, 75 and 90 mg $m^{-2}$, provided that no major toxicity was observed at the lower dose level before escalating the dose. Haematological toxicity was evaluated by daily blood sampling and toxic effects were registered and graded according to the World Health Organization (WHO) criteria. The

**Table 1** Patient characteristics

| Characteristic | Dose level of NOV (mg $m^{-2}$) 60 | 75 | 90 | P[a] |
|---|---|---|---|---|
| Total number of patients | 10 | 10 | 10 | |
| Age (years) | | | | |
| Median | 44 | 35 | 45 | NS |
| Range | 26–55 | 18–57 | 32–52 | |
| Sex (male/female) | 5/5 | 2/8 | 1/9 | |
| Previous therapy (score)[b] | | | | |
| Median | 1 | 1 | 1 | NS |
| Range | 0–4 | 0–3 | 0–4 | |
| Performance status | ≥ 80 | ≥ 80 | ≥ 80 | NS |
| Haematological parameters fore high-dose chemotherapy: | | | | |
| WBC (× $10^9$ $l^{-1}$) | | | | |
| Median | 5.420 | 5.070 | 6.915 | NS |
| Range | (3.100–7.930) | (3.480–10.400) | (3.940–11.690) | |
| Neutrophils (× $10^9$ $l^{-1}$) | | | | |
| Median | 3.800 | 3.400 | 4.435 | NS |
| Range | (1.770–7.030) | (2.150–9.360) | (2.360–8.700) | |
| Platelets (× $10^9$ $l^{-1}$) | | | | |
| Median | 300 | 311.5 | 269.5 | NS |
| Range | (192–433) | (172–450) | (195–369) | |
| Hb (mg $dl^{-1}$) | | | | |
| Median | 11.4 | 12.1 | 12.3 | NS |
| Range | (10.3–14.8) | (10.2–13.1) | (9.1–13.4) | |
| Bone marrow involvement | No | No | No | |
| CY-NOV + L-PAM interval (days) | | | | |
| Median | 26 | 28 | 26 | NS |
| Range | (19–39) | (23–54) | (20–67) | |
| Neutrophils before NOV (× $10^9$ $l^{-1}$) | | | | |
| Median | 4.675 | 3.5 | 4.585 | NS |
| Range | (0.99–19.730) | (1.350–14.6) | (2.32–10) | |
| Disease (BC/NHL) | 4/6 | 6/4 | 8/2 | |

[a]Kruskall–Wallis test. [b]Previous therapy score: 0, no previous chemoradiotherapy; 1, less than one course of standard-dose chemotherapy; 2, one complete course of standard-dose chemotherapy (six cycles); 3, more than one course of standard-dose chemotherapy; radiotherapy add one point to chemotherapy score.

three patient groups were well matched for factors that might influence haematopoietic recovery, as detailed in Table 1.

In order to evaluate the possible cytotoxic effect of residual plasma NOV on the graft, the plasma pharmacokinetics of NOV was evaluated by high-performance liquid chromatography (HPLC) in 13 patients and the correlation between the plasma concentration of the drug at the time of reinfusion and the haematological toxicity parameters was analysed. Furthermore, the in vitro sensitivity of haematopoietic progenitor cells to NOV was determined by measuring the surviving fraction of granulocyte–macrophage colony-forming units (CFU-GM) exposed to various drug concentrations in standard short-term (1 h) as well as in long-term (7 day) exposure tests.

### Patient characteristics and eligibility

Eighteen patients had metastatic breast cancer (BC) and 12 had intermediate or high-grade non-Hodgkin's lymphoma (NHL). Among the NHL patients, one was in first relapse and 11 were poor risk (as defined either by group 2–3 international index or by the presence of bulky disease) at diagnosis (The International Non-Hodgkin's Lymphoma Prognostic Factors Project, 1993). The main characteristics of the three groups of patients are listed in Table 1.

Eligibility criteria included age below 60 years, performance status ≥ 80% (Karnofsky) and normal heart, lung, liver and renal function. Patients with bone marrow involvement as determined by bilateral iliac biopsy, as well as patients with previous or concomitant neoplasia, diabetes mellitus or brain metastases, were excluded. All patients provided written, informed consent in keeping with institutional ethics committee guidelines.

### High-dose chemotherapy

Breast cancer patients received a four-step high-dose treatment as previously reported (Patrone et al, 1995), including: first, cyclophosphamide (CY) 6 g $m^{-2}$; second, NOV 60–90 mg $m^{-2}$ plus L-PAM 140–180 mg $m^{-2}$ and PBPC rescue; third, methotrexate 8 g $m^{-2}$ plus vincristine 1.4 mg $m^{-2}$; and fourth, etoposide 1.5 g $m^{-2}$ plus carboplatin 1.5 g $m^{-2}$ and PBPC rescue. NHL patients received similar treatment except for the metothrexate plus vincristine step which was omitted.

NOV was dissolved in saline and administered on day – 4 as a 4-h infusion, at the three dose levels of 60, 75 and 90 mg $m^{-2}$, with mild anti-emetic treatment. L-PAM was administered on day – 1, at 180 mg $m^{-2}$ with the lowest dose of NOV and at 140 mg $m^{-2}$ with the two highest dose levels. The drug was given undiluted in three divided doses, with intensive i.v. hydration and anti-emetic

© Cancer Research Campaign 1997

therapy consisting of dexamethasone and ondansetron. Cryopreserved PBPCs were reinfused on day 0, i.e. 96 h after NOV administration. No haematopoietic growth factors were administered after PBPC reinfusion.

### Haematopoietic progenitor cells

After CY administration patients received subcutaneous haematopoietic growth factors (rhGM-CSF or a sequential combination of rhGM-CSF and rh interleukin 3) until haematological recovery. PBPCs were collected by continuous flow leucapheresis starting when WBC and platelet counts reached $1.0 \times 10^9$ $l^{-1}$ and $50 \times 10^9$ $l^{-1}$ respectively. A median number of three aphereses (range 2–5) was required to collect the planned number of $CD34^+$ cells ($\geq 10 \times 10^6$ $kg^{-1}$), which we assumed would conveniently support two myeloblative cycles.

The apheretic product was cryopreserved in autologous plasma and 10% dimethylsulphoxide. Cells expressing the CD34 surface antigen were enumerated cytofluorimetrically with a Coulter Epics Profile 2 flow cytometer (Coulter, Hialeah, FL, USA) using the monoclonal antibody fluorescein isothiocyanate (FITC)-conjugated HPCA-2 (Becton-Dickinson, San Jose, CA, USA) (Siena et al, 1991).

### Pharmacological study

In 13 patients, blood samples for analysis of NOV concentrations were drawn before drug administration as well as at 1, 2 and 3 h during the infusion, at the end of the 4 h NOV infusion, and thereafter at 5, 15, 30, 45 and 60 min and at 2, 4, 6, 24, 48, 72 and 96 h. The samples, collected in heparinized tubes, were immediately placed on ice and then centrifuged at 1800 *g* for 10 min to separate plasma. Plasma samples, to which was added 10% (v/v) of 5% L-ascorbic acid in citrate buffer (0.1 M, pH 3.0), were frozen at −20°C until processing. Urine samples were collected over 4 h during infusion and then at successive intervals of 2, 4, 6, 24, 48, 72 and 96 h after the end of infusion. Each urine volume was recorded and an aliquot was frozen until analysis.

Analysis of NOV in plasma and urine was carried out by HPLC according to the method described by Peng et al (1982). Quantitation was done by the external method of analysis. Retention time for NOV was 5 min. Extraction efficacy from plasma and urine was about 90%. The detection limit of the method was 1 ng $ml^{-1}$.

Concentrations of NOV vs time were plotted on semilogarithmic graphs. Visual inspection of the post-infusion plasma concentration–time profiles suggested that the curves were triphasic in form. Therefore, the plasma concentration–time curves for each patient were analysed according to a three-compartment open model using a P-Pharm computer program (Simed France) on an IBM/IC computer. All plasma pharmacokinetics were fitted to the multiexponential equation: $C_p(t) = A \exp(-\alpha t) + B \exp(\beta t) + C \exp(-\gamma t)$, where $C_p(t)$ is the drug concentration at time $t$, $A$, $B$ and $C$ are constants and $\alpha$, $\beta$, and $\gamma$ are the apparent first-order elimination rate constants. The area under the concentration vs time curve (AUC), corrected for the duration of infusion, was calculated according to Freedman and Workman (1988). Mean residence time (MRT), steady-state volume of distribution ($V_{ss}$), total body clearance (Cl) and the elimination half-lives were calculated from standard pharmacokinetic equations (Gibaldi and Perrier, 1982). The renal clearance ($Cl_R$) was calculated using the equation $Cl_R = Du_t/AUC_t$, where $Du_t$ is the amount of NOV excreted in urine up to time $t$ after the infusion and $AUC_t$ is the area under the concentration–time curve calculated (trapezoidal rule) for the same time.

### Cytotoxicity assay

NOV cytotoxicity against haematopoietic progenitor cells was determined in a dose–surviving fraction curve by measuring the number of CFU-GM after in vitro exposure to various concentrations of the drug. PBPC samples from the apheretic product were exposed to various NOV concentrations that represented the plasma values measured during pharmacokinetic study, including the peak plasma concentrations and those measured at the time of progenitor cell reinfusion. The tests were performed after either a 1-h exposure time or a 7-day exposure time. NOV (Lederle-Cyanamid S.p.A.) was prepared in 0.9% sodium chloride solution immediately before use. For the 1-h exposure experiments cells were suspended in RPMI-1640 medium at $10^6$ $ml^{-1}$, incubated at 37°C with gentle shaking and then washed twice before clonogenic assay. For the 7-day exposure experiments the drug was directly mixed in clonogenic assay medium.

CFU-GM surviving fraction was determined in a modified short-term clonogenic assay (Lemoli and Gulati, 1993). Briefly, cells were plated in triplicate in 24-well tissue culture plates (Corning Costar Corporation, Cambridge, MA, USA). The culture medium consisted of 0.3 ml of Iscove MDM supplemented with

**Table 2** Haematological toxicity

| Parameter, median (range) | Dose level of NOV (mg $m^{-2}$) 60 | 75 | 90 | $P^a$ |
|---|---|---|---|---|
| Days with neutrophils $< 0.1 \times 10^9$ $l^{-1}$ | 7 (5–9) | 7 (5–9) | 6 (5–10) | NS |
| Days with neutrophils $< 0.5 \times 10^9$ $l^{-1}$ | 9.5 (9–15) | 11.5 (8–25) | 9.5 (8–18) | NS |
| Days to neutrophils $\geq 1 \times 10^9$ $l^{-1}$ | 16 (12–54) | 18 (15–28) | 18.5 (12–24) | NS |
| Days with platelets $< 10 \times 10^9$ $l^{-1}$ | 0 (0–1) | 0 (0–1) | 0.5 (0–4) | NS |
| Days with platelets $< 20 \times 10^9$ $l^{-1}$ | 2 (1–4) | 1.5 (0–6) | 2 (1–9) | NS |
| Units platelets transfused | 1 (0–2) | 1 (0–3) | 1 (0–3) | NS |
| Units packed RBCs transfused | 4 (2–8) | 3.5 (2–8) | 3 (1–6) | NS |
| Days with neutropenic fever < 38.5°C | 2 (0–7) | 1 (0–7) | 1 (0–9) | NS |
| Grade of mucositis | 2 (0–4) | 1 (0–4) | 1.5 (0–4) | NS |

[a]Kruskall–Wallis test.

**Table 3** Summary of NOV plasma pharmacokinetics

| | Dose level of NOV (mg $m^{-2}$) | | | |
|---|---|---|---|---|
| **Parameter** | **60** | **75** | **90** | **$P^a$** |
| Number of patients | 4 | 5 | 4 | |
| Peak level (μg $l^{-1}$) | 392 ± 19 | 602 ± 253 | 861 ± 270 | < 0.01 |
| $AUC_{0-\infty}$ (mg h $l^{-1}$) | 2.0 ± 0.3 | 2.9 ± 0.8 | 4.7 ± 1.3 | < 0.01 |
| MRT (h) | 61 ± 10 | 71 ± 25 | 63 ± 37 | NS |
| $T_{1/2\alpha}$ (min) | 15.0 ± 1.8 | 13.2 ± 1.8 | 14.4 ± 5.4 | NS |
| $T_{1/2\beta}$ (h) | 2.6 ± 0.3 | 2.0 ± 0.1 | 2.5 ± 0.8 | NS |
| $T_{1/2\gamma}$ (h) | 41 ± 7 | 48 ± 17 | 42 ± 16 | NS |
| 96 h level (μg $l^{-1}$) | 2.6 ± 1.1 | 3.4 ± 2.3 | 4.3 ± 1.8 | NS |
| Cl (l $h^{-1}$ $m^{-2}$) | 30 ± 4 | 28 ± 8 | 21 ± 7 | NS |
| $V_{SS}$ (l $m^{-2}$) | 1772 ± 139 | 1832 ± 459 | 1200 ± 618 | NS |
| $Cl_R$ (l $h^{-1}$) | 2.2 ± 0.8 | 2.0 ± 0.5 | 2.4 ± 1.4 | NS |

[a]Kendall correlation analysis. Abbreviations: AUC, area under plasma concentration vs time curve; MRT, mean residence time; CL, total-body clearance; $V_{SS}$, volume of distribution at steady-state; NS, not significant.

24% fetal calf serum (FCS, Hyclone Europe, UK), 0.8% bovine serum albumin (BSA, Stem Cell Technologies, Canada), $10^{-4}$ M 2-mercaptoethanol (Sigma Chemical, St Louis, MO, USA), 10% 5637 medium and 10 ng $ml^{-1}$ recombinant human granulocyte colony-stimulating factor (G-CSF, Amgen-Roche, Milan, Italy). Methylcellulose (Sigma) was added at a final concentration of 1.1%. CFU-GM colonies (≥ 50 cells) were scored after 7 days using an inverted microscope. The percentage of survival was calculated on the basis of three experiments as the ratio between the number of colonies surviving on cultures treated with NOV, 1 h or 7 days, and the number of colonies growing on control plates.

**Figure 1** Plasma concentration–time profiles of NOV following a 4 h intravenous infusion at the three dose levels studied. Points represent the mean of four patients at 60 mg $m^{-2}$ (■), five patients at 75 mg $m^{-2}$ (●) and four patients at 90 mg $m^{-2}$ (▲). —, 4 h infusion time

### Supportive care

All patients were supported in single or double rooms equipped with a high-efficiency particulate-air filtration unit until they achieved a neutrophil count ≥ 1.0 × $10^9$ $l^{-1}$. Transfusions of leucocyte-free packed red blood cells and single-donor platelets were administered for haemoglobin levels less than 9 g $dl^{-1}$ and platelet count less than 10 × $10^9$ $l^{-1}$. All patients received oral prophylaxis with ciprofloxacin and fluconazole and total parenteral nutrition when necessary.

### Response criteria

Before treatment patients were evaluated by means of physical examination, complete peripheral blood cell count and chemistry, chest radiography, chest and abdominal computerized tomographic scanning and bone marrow biopsy. In addition, breast cancer patients were evaluated by means of radionuclide bone scan and tumour markers (CA 15.3, CEA). Patients were restaged at the end of the sequential chemotherapy programme. Complete remission (CR) was defined as the disappearance of all measurable and assessable disease for at least 1 month. Partial remission (PR) was defined as a 50% or greater reduction in the product of the bidimensional measurements of all measured lesions with no new lesions and no lesions increasing in size. The persistent uptake of bone scan despite sclerosis of previous lytic lesions was defined as PR. Progressive disease (PD) was defined as a greater than 25% increase in tumour size or the appearance of any new lesion.

### Statistical analysis

As some data did not fit in with the normal frequency distribution, median ± SIQ (seminterquartile) were used where appropriate. Comparisons between groups were made using the Kruskall–Wallis test. Regression analysis was performed using the non-parametric Theil technique. The presence of any monotonic trend was assessed with the Kendall test (Hollander and Wolfe, 1974). Significance threshold was set at 0.05 level.

### RESULTS

### Haematological toxicity

The haematological recovery after high-dose CY was fast and complete in all 30 patients. The median duration of severe neutropenia (neutrophils < 0.5 × $10^9$ $l^{-1}$) was 6 days (range 2–10) and thrombocytopenia was severe (platelets < 20 × $10^9$ $l^{-1}$) in 20% of patients with a median duration of 2 days (range 1–4). The subsequent myelosuppressive course, NOV plus L-PAM, was administered after a median interval of about 4 weeks, 26 days (range 19–67). This interval was not significantly different in the three patient groups when discrete data were evaluated (Table 1).

© Cancer Research Campaign 1997

**Figure 2** Survival curves of CFU-GM incubated with various NOV concentrations in short-, 1 h (■) and long-term, 7 day (▲), in vitro tests. Points represent the mean ± s.e.m. of three experiments

The haematological toxicity parameters registered after NOV and L-PAM in the three groups of patients are listed in Table 2. Recovery from cytopenia was rapid and complete in all cases with a low requirement of single-donor platelet and packed red blood cell transfusions. In particular high-risk neutropenia (neutrophils $< 0.1 \times 10^9$ $l^{-1}$) lasted only about a week and high-risk thrombocytopenia (platelets $< 10 \times 10^9$ $l^{-1}$) was negligible. It is of note that no statistically significant differences were observed in the haematological recovery of the three patient groups. As a consequence, no statistical correlation was found between administered doses of NOV and the haematological parameters considered in Table 2.

### Peripheral progenitor cells

The myelosuppressive treatment with NOV and L-PAM was supported in all patients with peripheral blood progenitor cells collected in median 12 days (range 11–15) after high-dose CY. A median of three (range 2–5) leucaphereses were required to collect the planned number of CD34-positive cells to support two myelosuppressive courses. The median number of CD34-positive cells reinfused 96 h after NOV (day 0) was $12.4 \times 10^6$ $kg^{-1}$ (range 5.3–50.3), with no significant differences among the three dose levels of NOV ($P = 0.110$).

### Pharmacokinetics of high-dose NOV

Plasma disappearance curves for NOV at the three dose levels studied are shown in Figure 1. Post-infusion plasma NOV concentrations decayed in a triexponential fashion with an elimination mean half-life of 44.1 ± 17.1 h. Table 3 lists the mean pharmacokinetic parameters for each NOV dose level. The values for clearance (CL, CLR), $V_{ss}$, MRT, and half-lives were not significantly different between varying doses. A significant ($P < 0.01$) correlation was observed between administered doses and peak levels of NOV, as well as between administered doses and AUCs. In all patients, plasma NOV was still detectable on day 0, i.e. 96 h after drug administration. Measurement of NOV urinary excretion showed that 1.6%–6.7% (mean 3.6%) of the compound was excreted within 72 h, independently of the dose administered.

### Pharmacokinetics–pharmacodynamic relationships

In the 13 cases evaluated, NOV plasma pharmacokinetics did not correlate with toxicity over the three dose levels. In particular, no correlation was observed between $C_{max}$ and AUC and the duration of neutropenia and thrombocytopenia or platelet and red blood cell requirement or the degree of mucositis. It must also be noted that these toxicity parameters were not related with the NOV plasma concentration at 96 h, that is at the time of progenitor cell reinfusion.

### Sensitivity of CFU-GM to NOV

To verify the sensitivity of CFU-GM to NOV, samples of the apheretic products were incubated in vitro with various concentrations of the drug, as indicated by the pharmacokinetic analysis, and the survival fraction was evaluated by clonogenic assay. CFU-GM showed a high sensitivity to NOV both in short and long-term exposure tests (Figure 2). However, at low NOV concentrations (i.e. ≤ 5 ng $ml^{-1}$) the majority of cells escaped killing, even in long exposure tests. On the contrary, at high NOV concentrations nearly all cells underwent lethal damage, in particular with those similar to peak plasma concentrations as measured in vivo. In the intermediate part of the curves the survival fraction was dose dependent but, as expected, it showed a steeper course with longer exposures. It is of note that low doses of NOV, similar to the residual plasma concentrations measured at the time of progenitor cell reinfusion, had very low cytotoxic potential in both short- and long-term in vitro tests.

© Cancer Research Campaign 1997 

### Non-haematological toxicity

Non-haematological toxicity was graded according to the standard World Health Organization (WHO) system. No treatment-related deaths occurred. After high-dose CY no clinically relevant toxicities were registered.

At the time of NOV administration four patients (two at 75 and two at 90 mg $m^{-2}$) developed a febrile reaction, with chills, greater than 38°C that was rapidly reversed by hydrocortisone administration. Mucositis was observed in 23 patients, with a median duration of 7 days (range 2–24), and was severe enough to require analgesics and total parenteral nutrition in eight cases. The grading of mucositis was not found to be related to the administered dose level of NOV (Table 2).

Mild elevation of transaminases or bilirubin was observed in seven patients, WHO grade 1 or 2. No hepatic veno-occlusive disease was observed.

A 32-year-old patient with metastatic breast cancer had a 16% asymptomatic decrease in left ventricular ejection fraction (LVEF) over the baseline value 6 months after NOV 90 mg $m^{-2}$. At 12 months this patient developed reversible congestive heart failure and presented a further reduction in LVEF, 35% below the baseline value. During follow-up no other patients developed clinical evidence of cardiotoxicity.

Twenty patients presented neutropenic fever greater than 38.5°C for a median duration of 2.5 days (range 1–9). No severe infection with septicaemia was observed. The administered dose level of NOV was not correlated with the duration of neutropenic fever.

### Response to treatment

Among the 23 evaluable patients (seven patients had non-evaluable disease after induction chemotherapy or surgery) a high response rate to the full chemotherapy programme was observed at all dose levels of NOV. Among the 11 NHL patients, nine achieved CR (82%), one PR (9%) and one progressed through therapy with an overall response rate of 90.9%. In the metastatic breast cancer group the treatment induced CR in 7 out of 12 patients (58.3%) and PR in five (41.7%) with an overall response rate of 100%.

### DISCUSSION

A major end point of the present study was to evaluate whether NOV can be safely escalated up to 90 mg $m^{-2}$ when adequately supported by PBPCs. A second issue was to evaluate the optimal timing of PBPC reinfusion by determining the time interval required for plasma drug concentration to fall below the threshold of cytotoxicity against haematopoietic progenitors. The results indicate that high-dose NOV can be administered safely in the range of 60–90 mg $m^{-2}$, provided that adequate numbers of PBPCs are given. In fact, in our series high-risk neutropenia lasted on average 1 week and the duration of thrombocytopenia was negligible with no infectious or haemorrhagic complications and low transfusional requirement. No treatment-related mortality was observed and all patients completed the multistep high-dose chemotherapy as planned.

Different findings were reported by Attal and colleagues (1994) in a dose-finding study of high-dose NOV supported by bone marrow transplantation in 20 patients with refractory NHL. These authors reported a significant increase in haematological toxicity in a few patients receiving 90 mg $m^{-2}$ compared with patients receiving doses ranging from 15 to 75 mg $m^{-2}$, the mean duration of severe neutropenia (neutrophils < 0.5 × $10^9$ $l^{-1}$) being 31.7 days (s.d. 8) and 22 days (s.d. 6.5) respectively. Furthermore, they found a significant relationship between the duration of severe neutropenia and two pharmacokinetic parameters, $T_{1/2\gamma}$ and day 0 plasma concentration (192 h after NOV). Therefore, they concluded that NOV 90 mg $m^{-2}$ has a potential risk of unacceptable toxicity and that a minimum 8-day delay is required between NOV administration and graft reinfusion. In contrast, in our study haematological toxicity was the same at all dose levels of NOV and in particular the mean duration of severe neutropenia with 90 mg $m^{-2}$ was not significantly different when compared with the lower dose groups, that is 11.4 days (s.d. 3.7) vs 11.5 days (s.d. 3.8).

In our series, NOV pharmacokinetics did not differ from that described previously (Alberts et al, 1985; Ehninger et al, 1986; Van Belle et al, 1986; Richard et al, 1992; Canal et al, 1993; Attal et al, 1994). However, the duration of severe neutropenia was about 50% shorter than that reported by Attal and colleagues (1994). Furthermore, no delayed haematopoietic failures were observed over a median follow-up of 26 months (range 6–48) and no relationship was found between pharmacokinetic and haematological toxicity parameters in any of the 13 patients studied. To explain these discrepancies, we could speculate that different drugs were administered in association with NOV in the two studies, i.e. CY, BCNU and VP-16 vs L-PAM. However, we believe that a better explanation can be found by considering the different haematopoietic rescue. In fact, our patients were supported with PBPCs (median number of CD34-positive cells 12.4 × $10^6$ $kg^{-1}$, which is well above the known threshold limit for haematopoietic engraftment; Siena et al, 1991), whereas in the Attal et al's series bone marrow cells were used. It is well known (Siena et al, 1993; Martin, 1995) that PBPCs possess a higher bone marrow-repopulating capacity than bone marrow cells and allow a more rapid recovery of haematopoietic function. Furthermore, based on the prolonged plasma half-life of NOV, delays up to 8 days between drug administration and haematopoietic progenitor reinfusion are recommended (Mulder et al, 1989; Attal et al, 1994). In fact, at that point NOV plasma levels would be lower than 1 ng $ml^{-1}$, well below the threshold of 2.5 ng $ml^{-1}$ previously reported to produce a 50% inhibition of CFU-GM in long-term in vitro tests (Fountzilas et al, 1986). In the scheduling of high-dose therapy with NOV, however, graft timing is far from trivial, as anticipating the graft may allow shorter conditioning regimens, PBPC reinfusion before the onset of aplasia, fewer days of aplasia and reduced hospital stay. Taking into account these considerations, our patients were administered NOV over a 4-h infusion on day –4, and PBPCs were reinfused 96 h later when residual plasma NOV concentrations ranged from 1.5 to 5.5 ng $ml^{-1}$. All patients recovered from aplasia and no correlation was found between 96 h NOV plasma concentrations and measured haematological toxicity parameters. These clinical results strongly suggest that residual NOV at 96 h is devoid of any suppressive effect on reinfused progenitor cells. This is also supported by in vitro tests that were designed assuming that measured plasma concentrations reflect the bioavailability of the drug on target progenitor cells. In our experimental conditions, as evaluated by the CFU-GM assay, NOV concentrations up to 5 ng $ml^{-1}$ were incapable of inducing a significant cytotoxic effect on PBPCs. Contrary to what was previously reported, we were also able to confirm this observation in long-term exposure tests, that is a 7-day incubation, which might

© Cancer Research Campaign 1997

be considered a cytotoxicity assay that is more suitable for drugs with prolonged plasma half-life (Fountzilas et al, 1986). The absence of graft toxicity with 96 h earlier NOV administration has been recently reported by Stiff et al (1994) in ovarian cancer patients exposed to high-dose NOV, 75 mg $m^{-2}$ in three divided doses, and reinfused with autologous bone marrow.

In our patients non-haematological toxicity was low and substantially limited to mucositis. Mucositis is a frequent and important complication of high-dose NOV and L-PAM (Mulder et al, 1989; Ellis et al, 1990; Wallerstein et al, 1990; Bowers et al, 1993; Attal et al, 1994; Stiff et al, 1994; Patrone et al, 1995). Non-haematological toxicity was observed in 76% of patients in our series, and in a third (33%) of them it was of intermediate–high grade requiring parenteral nutrition and some analgesic treatment. However, mucositis was not associated with particularly severe infectious complications, healed promptly after resolution of neutropenia and did not result in any delay of planned chemotherapy. Unlike Attal's series with bone marrow rescue (Attal et al, 1994), there was no correlation between the NOV dose level and the severity of mucositis in our patients supported by PBPCS. The duration of neutropenia was uniformly short, and this may be a critical factor in determining the clinical evolution of mucositis, as suggested by the findings of Gabrilove et al (1988), who found a significant reduction in the incidence and severity of mucositis when neutrophil recovery after standard dose chemotherapy was accelerated by G-CSF. In our series, the only significant organ dysfunction was symptomatic heart failure observed in a breast cancer patient who presented a decline in LVEF 12 months after treatment at the 90-mg dose level. Dose-limiting cardiac toxicity with a maximum tolerated dose as low as 50 mg $m^{-2}$ was found when NOV was administered with high-dose thiotepa (Bowers et al, 1993). However, although decreases in LVEF or clinical signs of heart failure have been noticed, a dose-limiting heart toxicity has not been found in several studies using doses of NOV up to 80 mg $m^{-2}$ in combination with other potentially cardiotoxic agents such as CY and Ara-C (Mulder et al, 1989; Wallerstein et al, 1990; Feldman et al, 1993; Attal et al, 1994; Stiff et al, 1994). The single event observed in our study does not allow a conclusion to be made on the possible correlation between NOV dose levels and cardiotoxicity. It is hoped further information will be available from the prospective study we are presently running on the long-term survey of LVEF.

Both breast cancer and lymphoma patients had high response rates with the present multistep HD treatment. Although it is suggestive that NOV plays a significant role in this result, the present study was designed neither to compare NOV anti-tumour activity at the various dose levels nor to establish the relative anti-tumour activity of NOV in the sequential treatment. However, as our data demonstrate that PBPCs can overcome the myelosuppressive effect of doses of NOV up to 90 mg $m^{-2}$, and as containing haematological toxicity is a critical factor in reducing the overall morbidity of the treatment, we conclude that controlled studies with high-dose conditioning regimens including NOV up to 90 mg $m^{-2}$ can be planned for patients with breast cancer and non-Hodgkin's lymphoma.

### ACKNOWLEDGEMENTS

This work was partially supported by Ministero dell'Università e della Ricerca Scientifica e Tecnologica, Rome, Italy (60%) and by Consiglio Nazionale delle Ricerche, Rome, Italy (Progetto Finalizzato Applicazioni Cliniche della Ricerca Oncologica).

### REFERENCES

Alberts DS, Peng YM, Leigh S, Davis TP and Woodward DL (1985) Disposition of mitoxantrone in cancer patients. *Cancer Res* **45**: 1879–1884

Attal M, Canal P, Schlaifer D, Chatelut E, Dezeuze A, Huguet F, Payen C, Pris J and Laurent G (1994) Escalating dose of mitoxantrone with high-dose cyclophosphamide, carmustine, and etoposide in patients with refractory lymphoma undergoing autologous bone marrow transplantation. *J Clin Oncol* **12**: 141–148

Bowers C, Adkins D, Dunphy F, Harrison B, LeMaistre CF and Spitzer G (1993) Dose escalation of mitoxantrone given with thiotepa and autologous bone marrow transplantation for metastatic breast cancer. *Bone Marrow Transplant* **12**: 525–530

Canal P, Attal M, Chatelut E, Guichard S, Huguet F, Muller C, Schlaifer D, Laurent G, Houin G and Bugat R (1993) Plasma and cellular pharmacokinetics of mitoxantrone in high-dose chemotherapeutic regimen for refractory lymphomas. *Cancer Res* **53**: 4850–4854

Ehninger G, Proksch B, Heinzel G and Woodward DL (1986) Clinical pharmacology of mitoxantrone. *Cancer Treat* Rep **70**: 1373–1378

Ellis ED, William SF, Moormeier JA, Kaminer LS and Bitran JD (1990) A phase I–II study of high-dose cyclophosphamide, thiotepa and escalating dose of mitoxantrone with autologous stem cell rescue in patients with refractory malignancies. *Bone Marrow Transplant* **6**: 439–442

Feldman EJ, Alberts DS, Arlin Z, Ahmed A, Mittelman A, Baskind, Peng Y-M, Baier M and Plezia P (1993) Phase I clinical and pharmacokinetic evaluation of high-dose mitoxantrone in combination with cytarabine in patients with acute leukemia. *J Clin Oncol* **11**: 2002–2009

Fountzilas G, Ohnuma T, Rammos K, Mindich B and Holland FJ (1986) Comparison of mitoxantrone and ametantrone in human acute myelocytic leukemia cells in culture and in bone marrow granulocyte–macrophage progenitor cells. *Cancer Drug Delivery* **3**: 93–100

Freedman LS and Workman P (1988) When can the infusion period be safely ignored in the estimation of pharmacokinetic parameters of drugs in humans? *Cancer Chemother Pharmacol* **22**: 95–103

Frei E (1995) Pharmacologic strategies for high-dose chemotherapy. In *High-dose Cancer Therapy*. Armitage JO and Antman KH (eds), pp. 3–16. Williams & Wilkins: Baltimore

Frei E and Canellos GP (1980) Dose: A critical factor in cancer therapy. *Am J Med* **69**: 585–594

Gabrilove JL, Jakubosky A, Sher H, Stenberg C, Wong G, Grous J, Yagoda A, Fain K, Malcom MAS, Clarkson B, Oettgen HF, Alton K, Welte K and Souza L (1988) Effect of granulocyte colony-stimulating factor on neutropenia and associated morbidity due to chemotherapy for transitional-cell carcinoma of the urothelium. *N Engl J Med* **318**: 1414–1422

Gianni AM and Bonadonna G (1989) High-dose chemo-radiotherapy for sensitive tumors: Is sequential better than concurrent drug delivery? *Eur J Cancer Clin Oncol* **25**: 1027–1030

Gibaldi M and Perrier D (1982) *Pharmacokinetics*, 2nd edn. Dekker: New York

Hollander G and Wolfe KH (1974) *Nonparametric Statistical Methods*. John Wiley: New York

Lemoli RM and Gulati SC (1993) Effect of stem cell factor (c-kit ligand), granulocyte-macrophage colony-stimulating factor and interleukin3 on hematopoietic progenitors in human long-term bone marrow cultures. *Stem Cells* **11**: 435–444

Martin M (1995) High-dose chemotherapy for breast cancer: clinical advantages of autologous peripheral blood progenitor cells (PBPC) compared with autologous bone marrow transplantation (ABMT) *Ann Oncol* **6** (suppl. 4): S33–S37

Mulder POM, Sleijfer DT, Willemse PHB, De Vries EGE, Uges DRA and Mulder NH (1989) High-dose cyclophosphamide or melphalan with escalating doses of mitoxantrone and autologous bone marrow transplantation for refractory solid tumors. *Cancer Res* **49**: 4654–4658

Patrone F, Ballestrero A, Ferrando F, Brema F, Moraglio L, Valbonesi M, Basta P, Ghio R, Gobbi M, and Sessarego M (1995) Four-step high-dose sequential chemotherapy with double hematopoietic progenitor-cell rescue for metastatic breast cancer. *J Clin Oncol* **13**: 840–846

Peng YM, Ormberg D and Alberts DS (1982) Improved high-performance liquid chromatography of the new antineoplastic agents bisantrene and mitoxantrone. *J Chromatog Biomed Appl* **233**: 235–247

Richard B, Launay-Iliadis MC, Iliadis A, Just-Landi S, Blaise D, Stoppa AM, Viens P, Gaspard MH, Maraninchi D, Cano JP and Carcassonne Y (1992) Pharmacokinetics of mitoxantrone in cancer patients treated by high-dose chemotherapy and autologous bone-marrow transplantation. *Br J Cancer* **65**: 399–404

© Cancer Research Campaign 1997

Siena S, Bregni M, Brando B, Belli N, Ravagnani F, Gandola L, Stern AC, Lansdorp PM, Bonadonna G and Gianni AM (1991) Flow cytometry for clinical estimation of circulating hematopoietic progenitors for autologous transplantation in cancer patients. *Blood* **77**: 400–409

Siena S, Bregni M, Bonsi L, Strippoli P, Peccatori F, Magni M, Di Nicola M, Bagnara GP and Gianni AM (1993) Clinical implications of the heterogeneity of hematopoietic progenitors elicited in peripheral blood by anticancer therapy with cyclophosphamide and cytokine(s). *Stem Cells* **11** (suppl. 2): 72–75

Stiff PJ, McKenzie RS, Alberts DS, Sosman JA, Dolan JR, Rad N and McCloskey T (1994) Phase I clinical and pharmacokinetic study of high-dose mitoxantrone combined with carboplatin, cyclophosphamide, and autologous bone marrow rescue: high response rate for refractory ovarian carcinoma. *J Clin Oncol* **12**: 176–183

The International Non-Hodgkin's Lymphoma Prognostic Factors Project (1993) A predictive model for aggressive non-Hodgkin's lymphoma. *N Engl J Med* **329**: 987–994

Van Belle SJP, dE Planque MM, Smith IE, Van Oosterom AT, Schoemaker TJ, Deneve W and McVie JG (1986) Pharmacokinetics of mitoxantrone in human following single-agent infusion or intra-arterial injection therapy or combined-agent infusion therapy. *Cancer Chemother Pharmacol* **18**: 27–32

Von Hoff DD, Clark GM, Weiss GR, marshall MH, Buchok JB, Knight WA III and LeMaistre CF (1986) Use of in vitro dose response effect to select antineoplastics for high-dose or regional administration regimens. *J Clin Oncol* **4**: 1827–1834

Wallerstein R, Spitzer G, Dunphy F, Huan S, Hortobagyi G, Yau J, Buzdar A, Holmes F, Theriault R, Ewer M, LeMaistre CF, Dicke K and Deisseroth A (1990) A phase II study of mitoxantrone, etoposide, and thiotepa with autologous marrow support for patients with relapsed breast cancer. *J Clin Oncol* **8**: 1782–1788

 *© Cancer Research Campaign 1997*


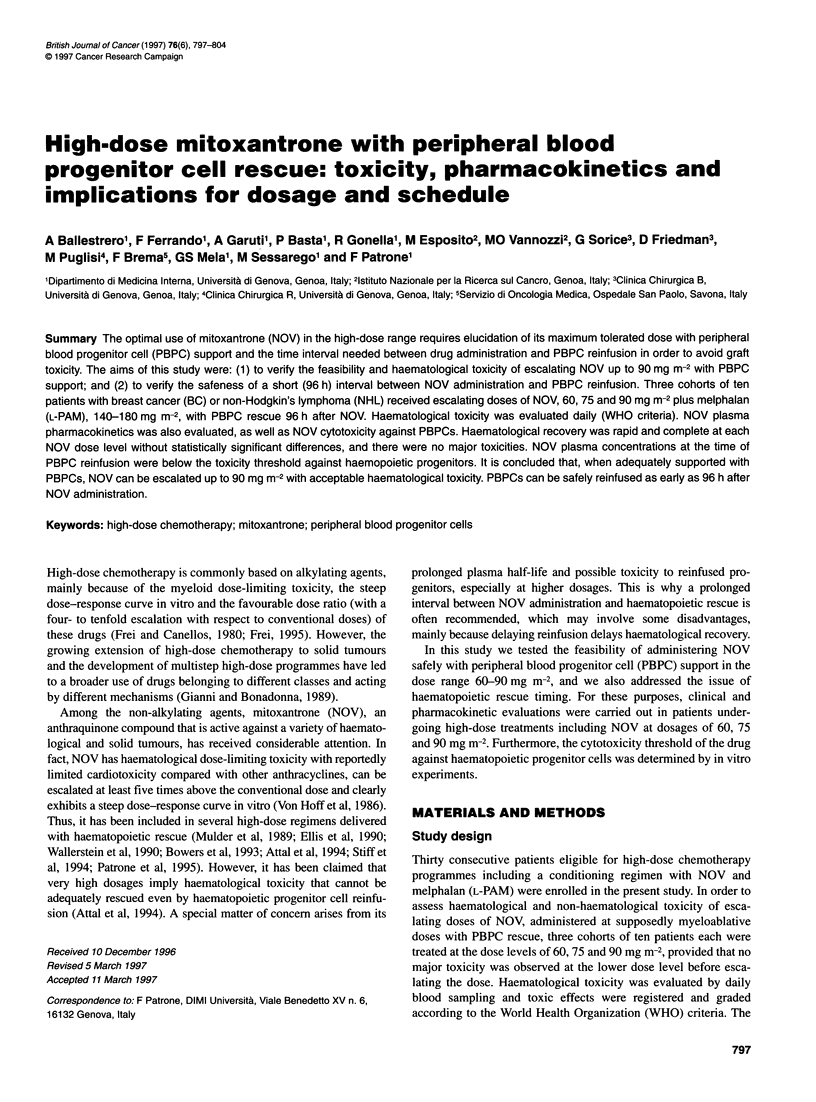

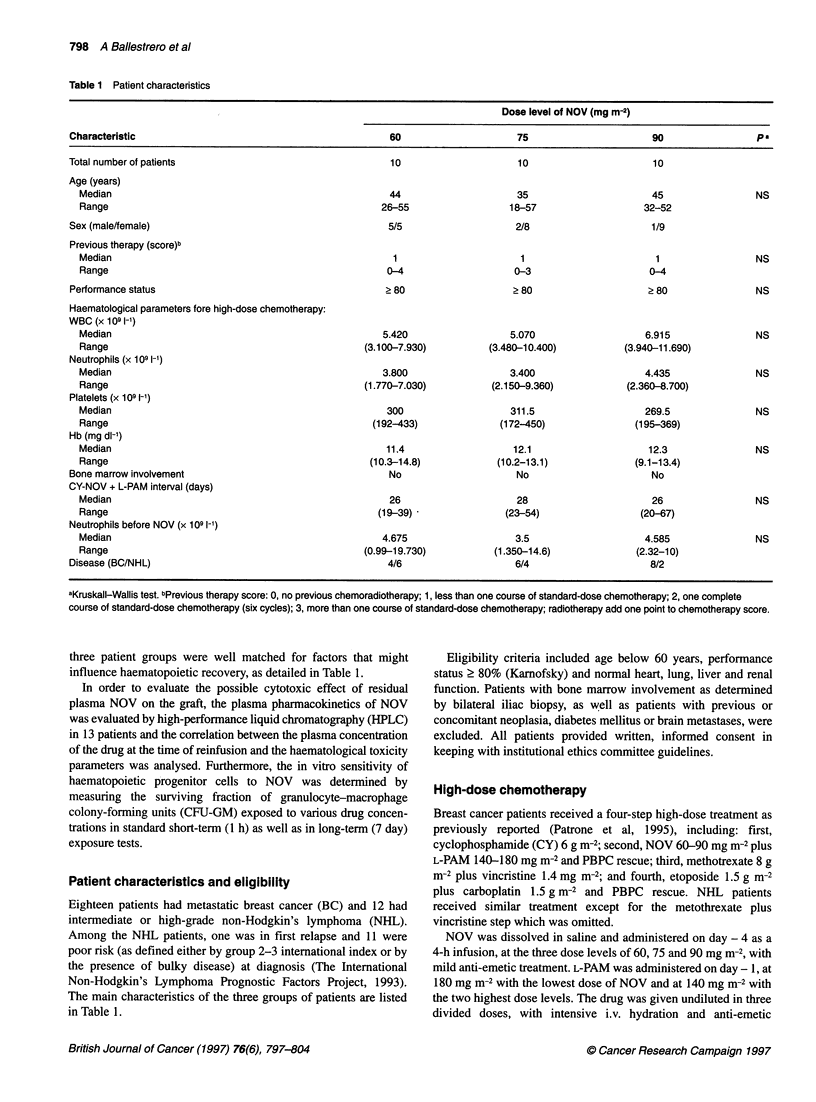

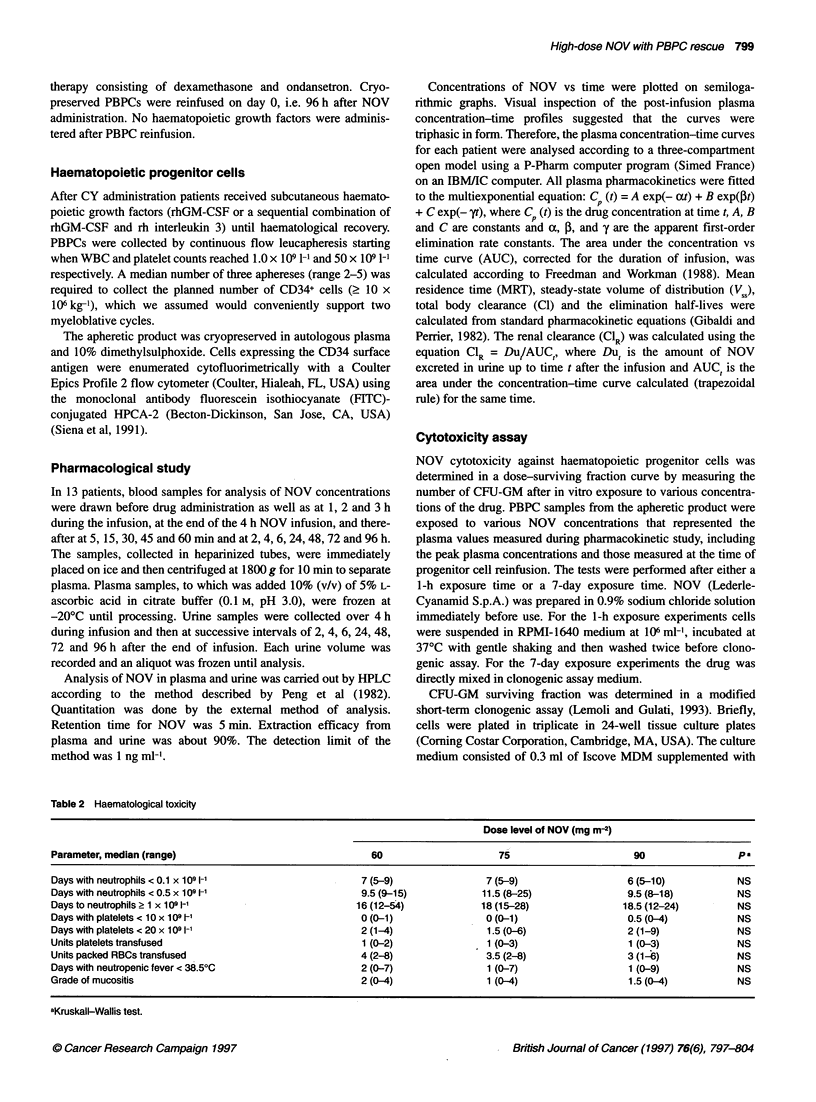

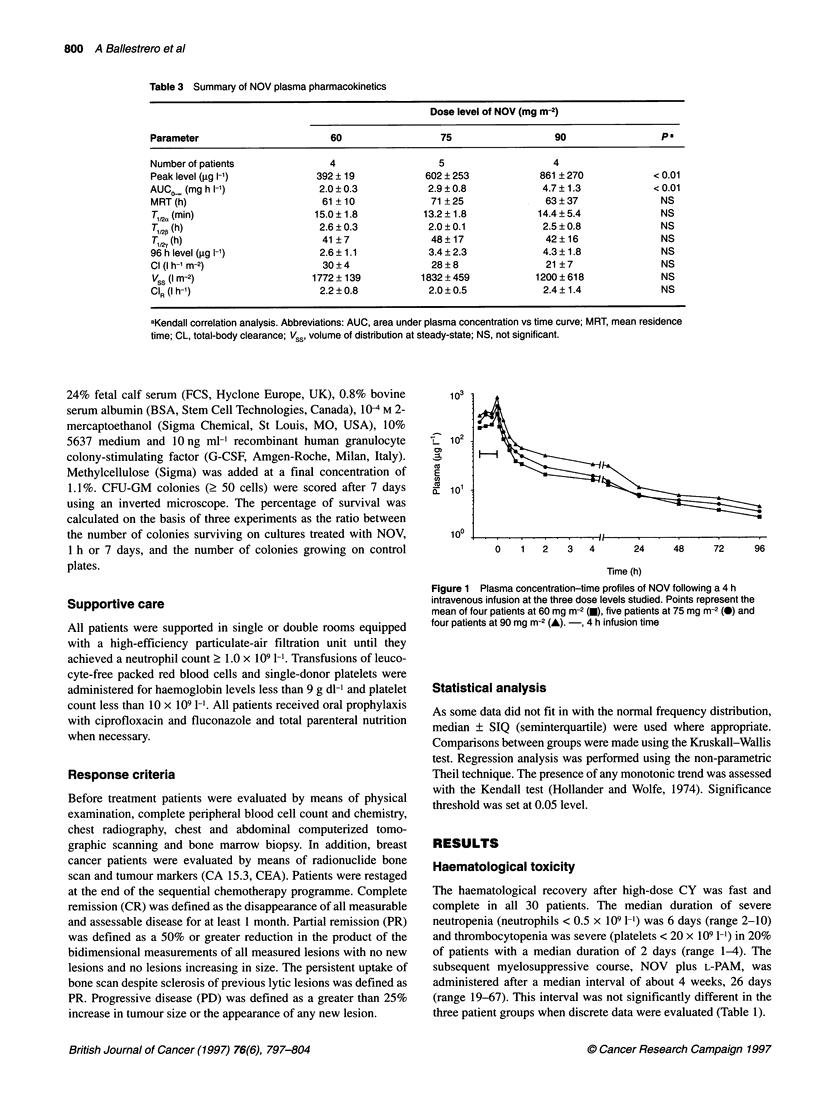

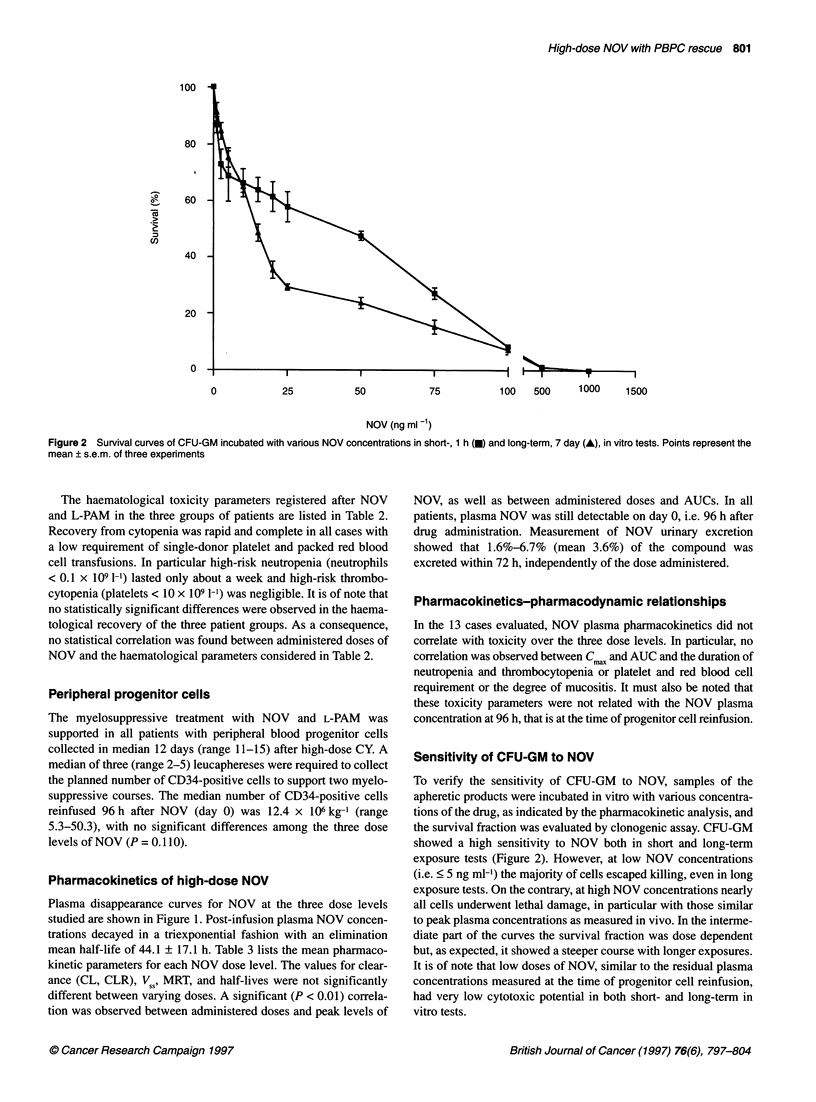

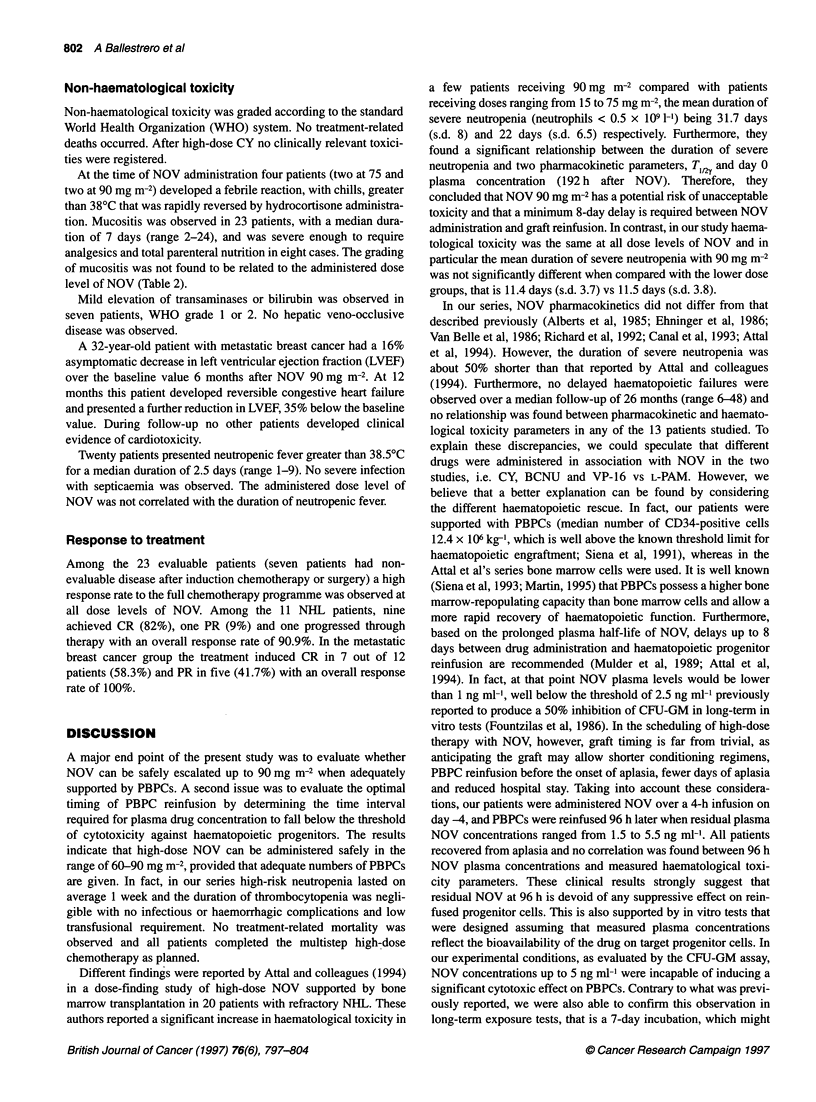

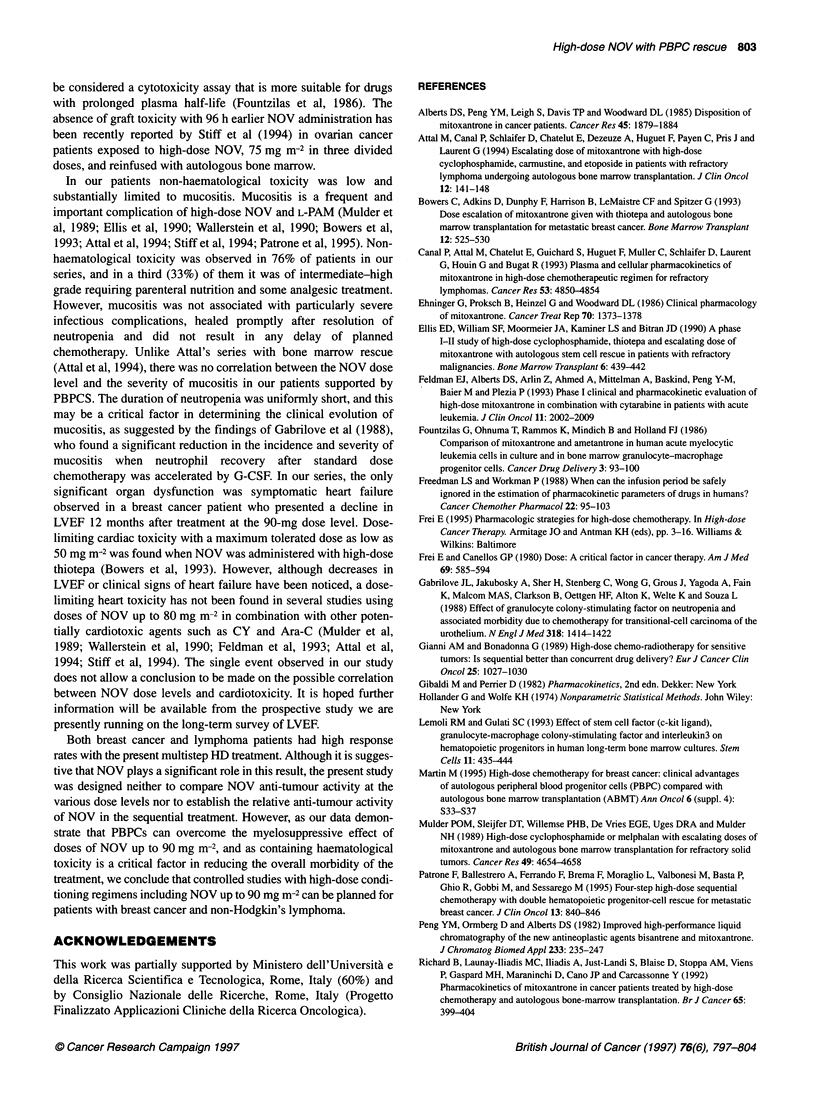

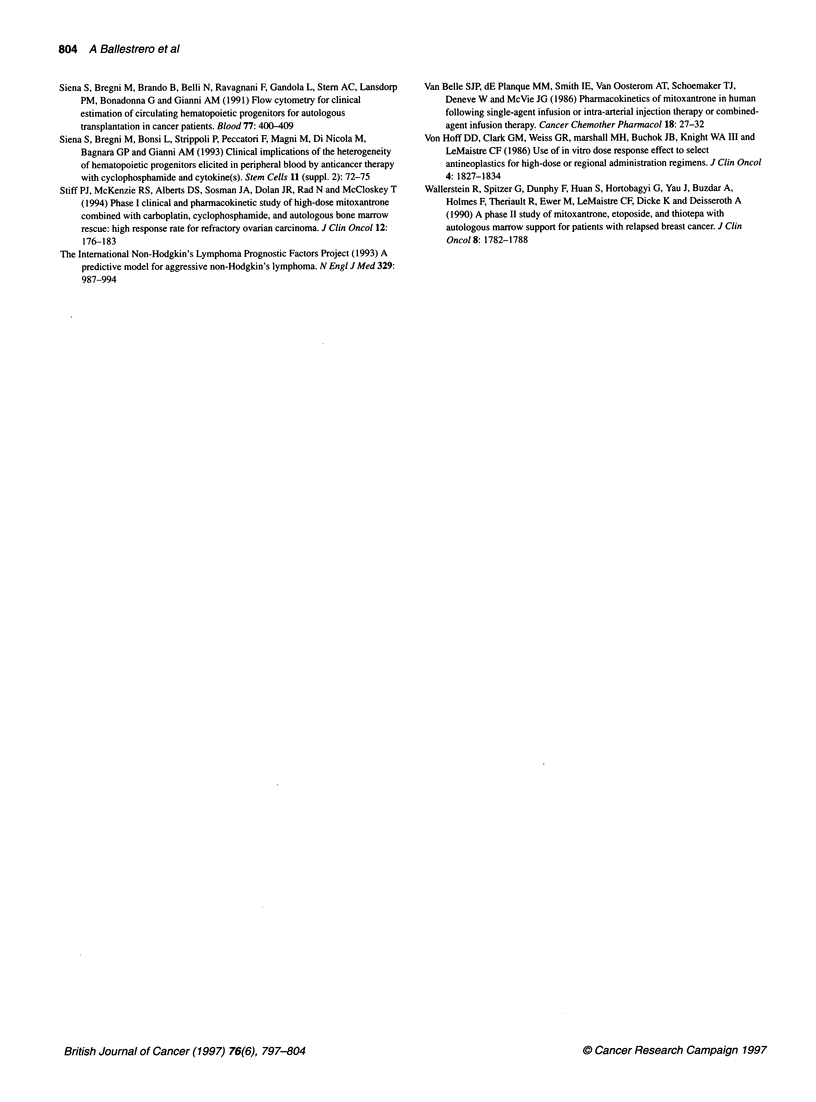


## References

[OCR_00749] Alberts D. S., Peng Y. M., Leigh S., Davis T. P., Woodward D. L. (1985). Disposition of mitoxantrone in cancer patients.. Cancer Res.

[OCR_00753] Attal M., Canal P., Schlaifer D., Chatelut E., Dezeuze A., Huguet F., Payen C., Pris J., Laurent G. (1994). Escalating dose of mitoxantrone with high-dose cyclophosphamide, carmustine, and etoposide in patients with refractory lymphoma undergoing autologous bone marrow transplantation.. J Clin Oncol.

[OCR_00762] Bowers C., Adkins D., Dunphy F., Harrison B., LeMaistre C. F., Spitzer G. (1993). Dose escalation of mitoxantrone given with thiotepa and autologous bone marrow transplantation for metastatic breast cancer.. Bone Marrow Transplant.

[OCR_00769] Canal P., Attal M., Chatelut E., Guichard S., Huguet F., Muller C., Schlaifer D., Laurent G., Houin G., Bugat R. (1993). Plasma and cellular pharmacokinetics of mitoxantrone in high-dose chemotherapeutic regimen for refractory lymphomas.. Cancer Res.

[OCR_00775] Ehninger G., Proksch B., Heinzel G., Woodward D. L. (1986). Clinical pharmacology of mitoxantrone.. Cancer Treat Rep.

[OCR_00779] Ellis E. D., Williams S. F., Moormeier J. A., Kaminer L. S., Bitran J. D. (1990). A phase I-II study of high-dose cyclophosphamide, thiotepa and escalating doses of mitoxantrone with autologous stem cell rescue in patients with refractory malignancies.. Bone Marrow Transplant.

[OCR_00787] Feldman E. J., Alberts D. S., Arlin Z., Ahmed T., Mittelman A., Baskind P., Peng Y. M., Baier M., Plezia P. (1993). Phase I clinical and pharmacokinetic evaluation of high-dose mitoxantrone in combination with cytarabine in patients with acute leukemia.. J Clin Oncol.

[OCR_00791] Fountzilas G., Ohnuma T., Rammos K., Mindich B., Holland J. F. (1986). Comparison of mitoxantrone and ametantrone in human acute myelocytic leukemia cells in culture and in bone marrow granulocyte-macrophage progenitor cells.. Cancer Drug Deliv.

[OCR_00797] Freedman L. S., Workman P. (1988). When can the infusion period be safely ignored in the estimation of pharmacokinetic parameters of drugs in humans?. Cancer Chemother Pharmacol.

[OCR_00807] Frei E., Canellos G. P. (1980). Dose: a critical factor in cancer chemotherapy.. Am J Med.

[OCR_00811] Gabrilove J. L., Jakubowski A., Scher H., Sternberg C., Wong G., Grous J., Yagoda A., Fain K., Moore M. A., Clarkson B. (1988). Effect of granulocyte colony-stimulating factor on neutropenia and associated morbidity due to chemotherapy for transitional-cell carcinoma of the urothelium.. N Engl J Med.

[OCR_00819] Gianni A. M., Bonadonna G. (1989). High dose chemo-radiotherapy for sensitive tumors: is sequential better than concurrent drug delivery?. Eur J Cancer Clin Oncol.

[OCR_00830] Lemoli R. M., Gulati S. C. (1993). Effect of stem cell factor (c-kit ligand), granulocyte-macrophage colony stimulating factor and interleukin 3 on hematopoietic progenitors in human long-term bone marrow cultures.. Stem Cells.

[OCR_00844] Mulder P. O., Sleijfer D. T., Willemse P. H., de Vries E. G., Uges D. R., Mulder N. H. (1989). High-dose cyclophosphamide or melphalan with escalating doses of mitoxantrone and autologous bone marrow transplantation for refractory solid tumors.. Cancer Res.

[OCR_00850] Patrone F., Ballestrero A., Ferrando F., Brema F., Moraglio L., Valbonesi M., Basta P., Ghio R., Gobbi M., Sessarego M. (1995). Four-step high-dose sequential chemotherapy with double hematopoietic progenitor-cell rescue for metastatic breast cancer.. J Clin Oncol.

[OCR_00857] Peng Y. M., Ormberg D., Alberts D. S., Davis T. P. (1982). Improved high-performance liquid chromatography of the new antineoplastic agents bisantrene and mitoxantrone.. J Chromatogr.

[OCR_00862] Richard B., Launay-Iliadis M. C., Iliadis A., Just-Landi S., Blaise D., Stoppa A. M., Viens P., Gaspard M. H., Maraninchi D., Cano J. P. (1992). Pharmacokinetics of mitoxantrone in cancer patients treated by high-dose chemotherapy and autologous bone marrow transplantation.. Br J Cancer.

[OCR_00881] Siena S., Bregni M., Bonsi L., Strippoli P., Peccatori F., Magni M., Di Nicola M., Bagnara G. P., Massimo Gianni A. (1993). Clinical implications of the heterogeneity of hematopoietic progenitors elicited in peripheral blood by anticancer therapy with cyclophosphamide and cytokine(s).. Stem Cells.

[OCR_00875] Siena S., Bregni M., Brando B., Belli N., Ravagnani F., Gandola L., Stern A. C., Lansdorp P. M., Bonadonna G., Gianni A. M. (1991). Flow cytometry for clinical estimation of circulating hematopoietic progenitors for autologous transplantation in cancer patients.. Blood.

[OCR_00887] Stiff P. J., McKenzie R. S., Alberts D. S., Sosman J. A., Dolan J. R., Rad N., McCloskey T. (1994). Phase I clinical and pharmacokinetic study of high-dose mitoxantrone combined with carboplatin, cyclophosphamide, and autologous bone marrow rescue: high response rate for refractory ovarian carcinoma.. J Clin Oncol.

[OCR_00899] Van Belle S. J., de Planque M. M., Smith I. E., van Oosterom A. T., Schoemaker T. J., Deneve W., McVie J. G. (1986). Pharmacokinetics of mitoxantrone in humans following single-agent infusion or intra-arterial injection therapy or combined-agent infusion therapy.. Cancer Chemother Pharmacol.

[OCR_00907] Von Hoff D. D., Clark G. M., Weiss G. R., Marshall M. H., Buchok J. B., Knight W. A., LeMaistre C. F. (1986). Use of in vitro dose response effects to select antineoplastics for high-dose or regional administration regimens.. J Clin Oncol.

[OCR_00912] Wallerstein R., Spitzer G., Dunphy F., Huan S., Hortobagyi G., Yau J., Buzdar A., Holmes F., Theriault R., Ewer M. (1990). A phase II study of mitoxantrone, etoposide, and thiotepa with autologous marrow support for patients with relapsed breast cancer.. J Clin Oncol.

